# Crossed and Locked Quotes in a Multi-Market Simulation

**DOI:** 10.1371/journal.pone.0151096

**Published:** 2016-03-09

**Authors:** Andrew Todd, Peter Beling, William Scherer

**Affiliations:** Department of Systems and Information Engineering, School of Engineering and Applied Science, University of Virginia, Charlottesville, VA, United States of America; East China University of Science and Technology, CHINA

## Abstract

Financial markets are often fragmented, introducing the possibility that quotes in identical securities may become crossed or locked. There are a number of theoretical explanations for the existence of crossed and locked quotes, including competition, simultaneous actions, inattentiveness, fee structure and market access. In this paper, we perform a simulation experiment designed to examine the effect of simple order routing procedures on the properties of a fragmented market consisting of a single security trading in two independent limit order books. The quotes in the two markets are connected solely by the routing decision of the market participants. We report on the health of the consolidated market as measured by the duration of crossed and locked states, as well as the spread and the volatility of transaction prices in the consolidated market. We aim to quantify exactly how the prevalence of order routing among a population of market participants affects properties of the consolidated market. Our model contributes to the zero-intelligence literature by treating order routing as an experimental variable. Additionally, we introduce a parsimonious heuristic for limit order routing, allowing us to study the effects of both market order routing and limit order routing. Our model refines intuition for the sometimes subtle relationships between the prevalence of order routing and various market measures. Our model also provides a benchmark for more complex agent-based models.

## Introduction

Financial market participants face a competitive and diverse multi-venue financial system. Brokers and traders monitor a multitude of market feeds, which they rely upon to manage their trading operations. The number and variety of venues increases the complexity of trading decisions. Orders may be routed to a large number of exchanges, which may vary widely with respect to the trading platform, fees and rebates, liquidity and transparency.

Despite the complexity of the trading landscape, prices across venues should largely agree; the law of one price in financial markets should hold so far as traders can identify and act on arbitrage opportunities [1]. However, arbitrage is not the only type of strategic behavior that aligns quotes and prices across venues. Market participants with access to multiple trading venues route new orders and revise existing orders according to the state of the market as a whole.

Understanding the interaction of competing markets is of critical importance for policy-makers. Zero-intelligence (ZI) models have proven useful in the analysis of individual limit order book markets, but have not been widely applied to study multi-market systems. We propose an extension to the standard ZI model to a multi-market setting by introducing order routing as an experimental variable. Prior to discussing the model in detail, we first discuss the explanatory power of ZI models, and discuss how our model in particular contributes to the existing literature.

ZI models provide explanations by focusing on the deterministic mechanisms at play within a given system. The approach differs from the usual methods of economic theorizing, where the focus is on the strategic behavior of agents. Not only do ZI models differ from the standard game-theoretic models of economics, but they also differ from agent-based models. While agent-based models often do include realistic implementations of market mechanisms, their focus is usually on local interactions and the evolution of strategies or agent compositions. While ZI models may be viewed as simple agent based models, their mode of explanation is fundamentally different.

ZI models provide a mechanism-based explanation, with the idea that “proper explanations should detail the cogs and wheels of the causal process through which the outcome to be explained was brought about” [2]. In a ZI model of financial markets, however, we admit defeat with respect to the possibility of modeling all of the possible strategies at play. Instead, we provide a simple random model of aggregate order flow. The order flow model is a maximum entropy distribution across allowable actions within the market. With a model of order flow in place, we can then isolate the effects of the mechanisms, which in our case, are the limit order book and the order routing procedures.

ZI models have a long history, dating back to work by Becker [3]. More recently, ZI models have demonstrated remarkable predictive power when order flow parameters are fit to data [4]. ZI models have also been used to theorize about fundamental properties of markets such as liquidity and price impact [5]. ZI models have also proven useful for the evaluation of competing realize variance estimators [6]. In addition, ZI models have been used to compare the allocative efficiency of alternative market structures and latencies [7].

In this work, we present a two-market model where order routing is the only mechanism that integrates prices across markets. Our experimental design varies the prevalence of limit order and market order routing. Agents either have access to both markets, in which case they may route their orders, or have access to only one market, in which case their orders may trade through better quotes. The primary contribution is the study of the prevalence of order routing on various market measures, including spread, volatility and crossed or locked states. Prior work does not focus on order routing as the experimental variable. We present new results based on a unique experimental design. Along these lines, the contribution of the paper includes development of intuition with regards to the above relationships. Additionally, we introduce a parsimonious heuristic for limit order routing, which allows us to study the effects of both market order routing and limit order routing. Finally, our model provides baseline results with which to compare more complex models.

### Limit Order Book

A limit order book is a continuously evolving record of outstanding orders to buy or sell a financial product. When a limit order is submitted to an exchange it is either matched with another outstanding order, queued in the limit order book, or rejected. Limit orders have a price, quantity and side, which are the primary attributes that govern how they are processed. The price and quantity of a limit order are restricted to multiples of the tick size and lot size, respectively. The tick size is the minimum price increment (e.g. a penny). The lot size is the minimum quantity that may be traded in a single transaction (e.g. 100 shares). A limit order may be rejected if it does not adhere to these restrictions. A limit order only executes if there exists an appropriately priced order on the opposite side of the market. Limit orders are queued in the book if they cannot be executed. Queued orders may be modified or canceled.

The queued orders establish the best bid and ask prices. Market participants seeking to trade immediately will buy at the best ask price and sell at the best bid price. The queued orders are executed according to rules of precedence. The primary rule of precedence is price. The secondary rule of precedence is time. The limit order book is essentially a FIFO queue in which orders are executed based on price and origination time. However, orders may also be matched on a pro-rata basis, and matching algorithms, in general, may vary across exchanges and products.

Generally speaking, exchanges offer limit order book functionality beyond the basics required to operate a continuous double auction. Limit orders may have additional attributes that govern specific aspects of their display and execution. For example, some exchanges offer the ability to place hidden orders. Other attributes might control details of execution (e.g. immediate-or-cancel, fill-or-kill or all-or-none). In the United States, exchanges have introduced order types that are tied to specifics of the market structure (e.g. orders pegged to the national best bid and offer).

For modeling purposes, we choose to focus on only the core functionality required for the operation of a continuous double auction. Going forward, we make a few simplifying assumptions. First, the only order types will be market orders or limit orders with unit quantity. Unit order quantities eliminate the need for logic to handle partial executions. Instructions may be placed for the creation of a new order or the cancelation of an existing order.

**Fig 1:**
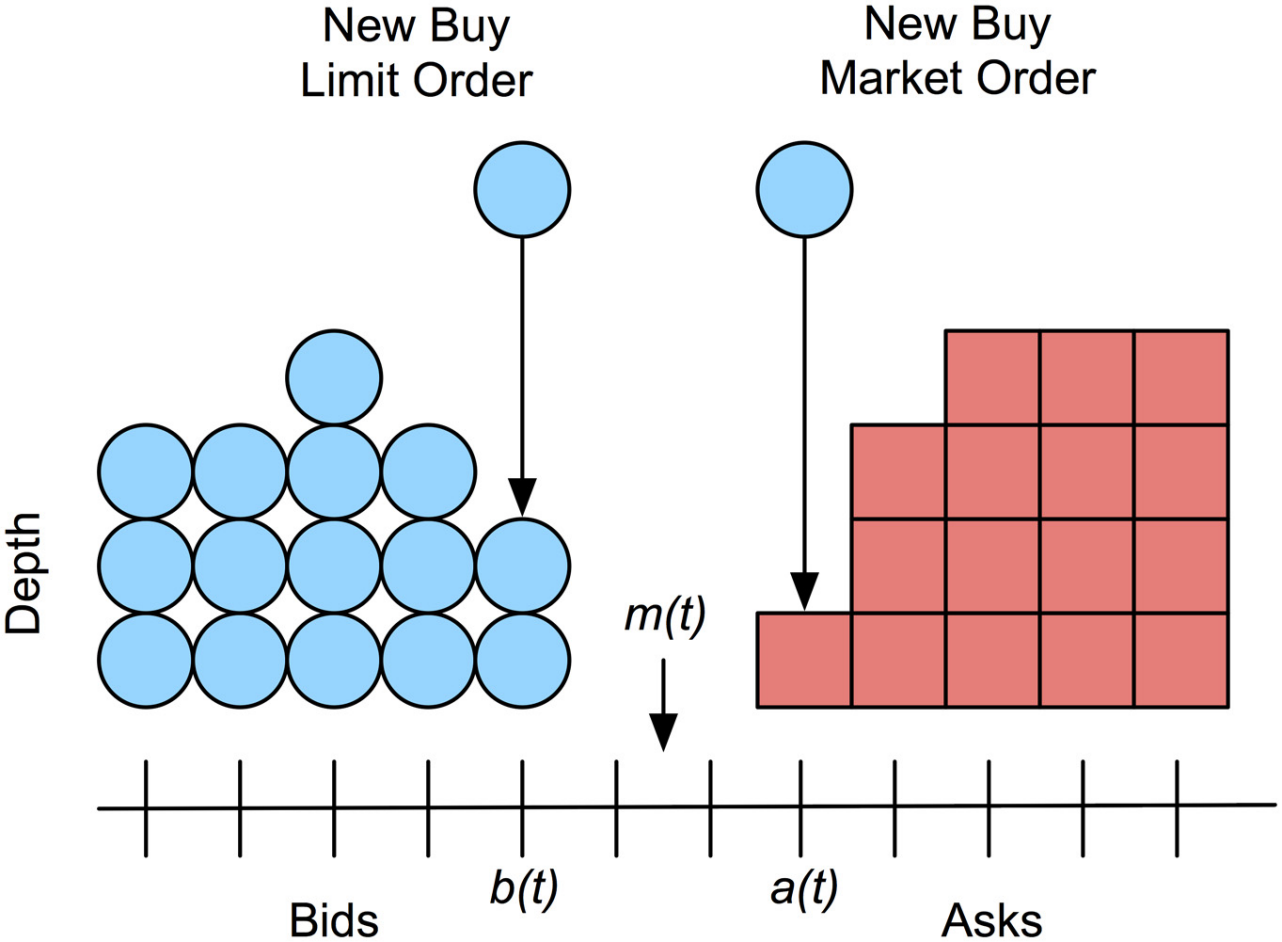
Limit order book diagram. A new buy limit order arrives at price *b*(*t*) increasing the depth from 2 to 3. A new buy market order executes at the best ask increasing the spread and changing the mid-quote.

Fig 1 depicts the evolution of the limit order book as a new limit order to buy and a market order to sell arrive. The best bid and ask at time *t* are denoted *b*(*t*) and *a*(*t*), respectively. In the figure, a buy limit order increases the depth at the best bid. Depth is the aggregate quantity available at a particular price. A market order to buy consumes the remaining depth at *a*(*t*), subsequently increasing both the spread, *a*(*t*) − *b*(*t*), and mid-quote, (*a*(*t*) + *b*(*t*))2.

Limit order books, and financial time-series more generally, may exhibit a number of statistical features, commonly referred to as stylized fact [8]. The stylized facts of an order book vary across securities and markets, and may be effected by external factors, such as wider market volatility. For the purposes of our analysis, the primary focus is on the interaction of bid and ask quotes in a two-market system. However, we also examine transaction price volatility. While there are a number of other stylized facts of financial markets, we feel that we have selected those most relevant to the goal of the analysis.

### Market Fragmentation

Many countries have opened the door for competition in their financial markets. In the United States, equity trades are distributed across 11 exchanges and over 40 alternative trading systems. There are also over 200 broker-dealers who internalize client orders [9]. In Europe, the Markets in Financial Instruments Directive (MiFID) also increased competition in equity trading [10]. Securities also trade in other countries and in different currencies. Competition among exchanges can benefit investors, but market fragmentation underlies a number of controversial practices related to high frequency trading [11]. Understanding how individual markets are linked together is critical for our ability to assess the quality and function of the overall market [12].

In a fragmented market, traders must determine how attractive each venue is for trading. When a market participant makes an investment decision that decision is translated into a stream of orders, which may be routed to a number of available exchanges. Market participants may also trade on alternative venues, such as dark pools. Decision-makers must weigh a number of factors, such as price, liquidity, fees and rebates. Orders that are not appropriately routed may trade through better quotes.

A trade-through is a transaction that executes at quotes that are inferior to quotes simultaneously available in another market. For example, if *b*_1_(*t*) > *b*_2_(*t*) and a market sell order executes in market 2, a trade-through occurs. Given the opportunity, a rational market participant would prefer to sell at a higher price and route the order to market 1 since it has superior quotes. The United States has regulations that are designed to protect retail order flow. For example, Reg NMS (Rule 610.d and Rule 611) establishes specific rules regarding trade-throughs and the display of crossed or locked quotes. Many markets, however, have no such restrictions and quotes across markets are connected only by the behavior of market participants.

**Fig 2:**
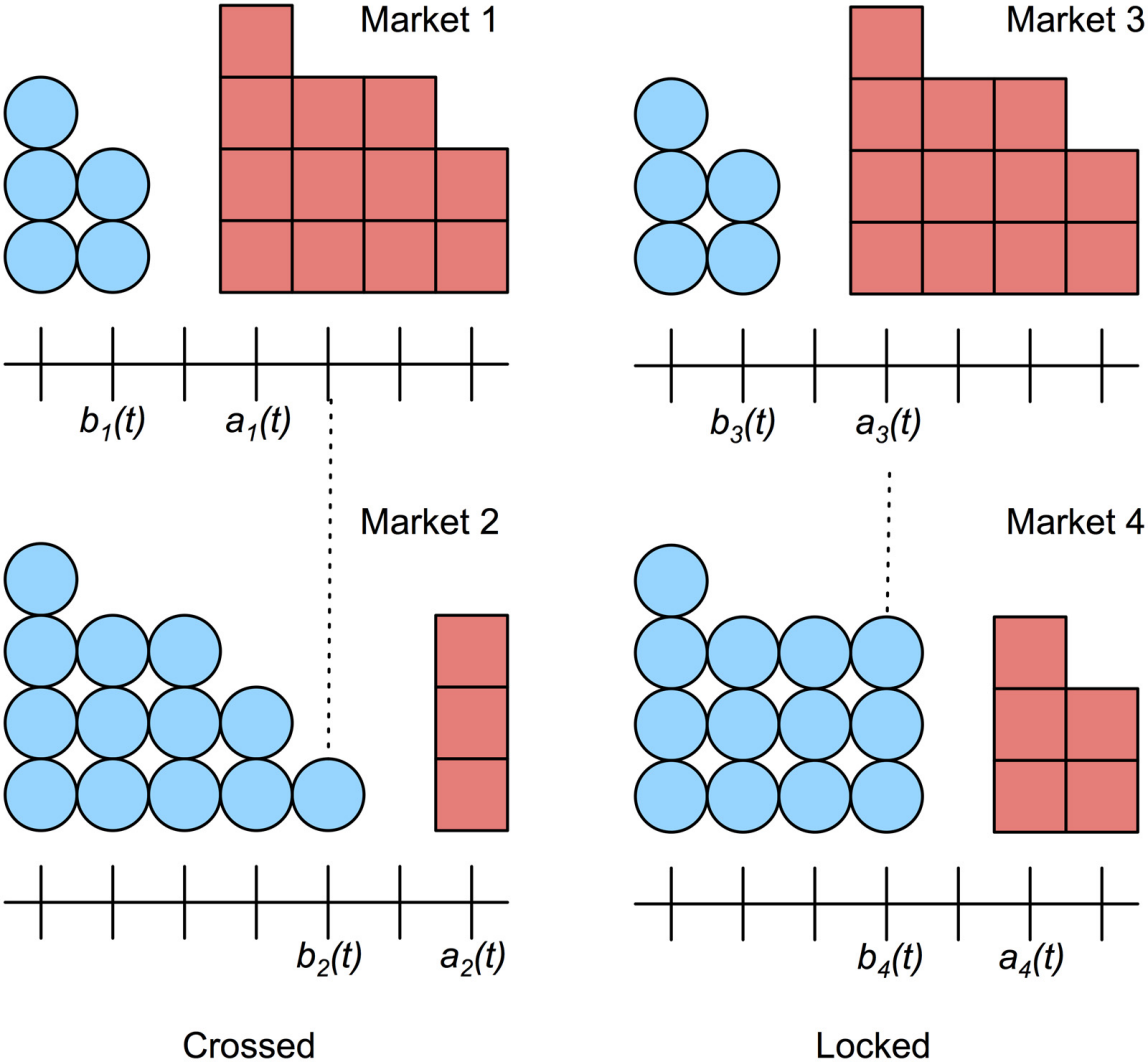
Crossed and locked quotes. Market 1 and Market 2 are crossed since *a*_1_(*t*) < *b*_2_(*t*). Market 3 and Market 4 are locked since *a*_3_(*t*) = *b*_4_(*t*).

Market participants may also seek to exploit arbitrage opportunities. An opportunity for instantaneous profit exists if a bid price in one market is greater than an ask price in another. The size of the arbitrage opportunity is constrained by the depth available at each price. In a two-market setting, markets are crossed if *b*_1_(*t*) > *a*_2_(*t*) or *b*_2_(*t*) > *a*_1_(*t*). Markets are locked if *b*_1_(*t*) = *a*_2_(*t*) or *b*_2_(*t*) = *a*_1_(*t*) (Fig 2). Crossed markets represent a theoretical arbitrage opportunity, which may be difficult to exploit in practice.

Crossed and locked markets arise naturally in fragmented markets. Cao, Ghysels and Hatheway (2000) find locked and crossed markets in the pre-opening period are used by dealers to signal information [13]. Shkilko, Van Ness and Van Ness (2008) find that the national best bid and offer is locked or crossed roughly 10.5% of the time for NASDAQ stocks and approximately 4% of the time for NYSE stocks and cite a number of potential causes, including simultaneous actions, lack of coordination, infrastructure issues, as well as differential fees and rebates [14]. Garvey and Murphy (2006) find markets locked 14% of the time and crossed 0.5% of the time for a sample of 20 NASDAQ stocks and conclude that institutional traders should be able to exploit arbitrage opportunities presented by crossed markets [15].

In this paper, we quantify the relationship between order routing and the time markets spend in cross or locked states. We also report the effect of order routing on other statistical properties of the consolidated limit order book.

## Methods

Our methodology is motivated by the zero-intelligence approach popularized by Gode and Sunder (1993) and applied widely in both economics and finance [16]. The key notion is that the mechanics of the system, in our case the limit order book and the order routing algorithms, are of utmost importance, and potentially dominate specifications of strategic behavior. From an agent-based modeling perspective, the model is populated with simple randomly behaving agents that interact within a given market structure.

The approach has a number of origins and motivations. In our case, we seek to avoid making detailed assumptions regarding the strategic behavior of agents (beyond routing) since that behavior varies widely and is difficult to model. More generally, we seek a balance between the idea of “keeping it simple” and “keeping it descriptive” [17, 18]. Specifically, our model builds on a line of work that models order arrivals and cancellations as a random process [19–24]. We adopt a model of order flow based on Smith et al (2003).

### Single-Market Model

In the single market setting, the limit order book is modeled as a multi-dimensional Markov process (Eq (1)), where negative components represent bid depth and positive components represent ask depth.

$$X_{i}\left(t\right)=\left\{X_{i}^{1}\left(t\right),\ldots X_{i}^{n}\left(t\right)\right\}\in Z^{n}$$ (1)

The best bid and ask prices at time *t* in market *i*, as mentioned previously, are denoted *b*_*i*_(*t*) and *a*_*i*_(*t*), respectively. If the bid side of the market is cleared out the best bid is set to 0. If the ask side of the market is cleared out, the best ask is set to *n*. Any given state transition consists of the addition or subtraction of a vector *e*^*j*^, which is the *j*th row of an identity matrix of size *n*, i.e. for state $x\in Z^{n}$, all transitions are for the form *x* → *x* ± *e*^*j*^. Each transition represents the addition or cancelation of a limit order or the execution of a market order.

For convenience, the process is defined by the arrival rates of limit orders, market orders and cancellations. Limit orders arrive uniformly at random relative to the opposite quotes according to a band *ν*. Limit orders arrive at a rate of *ℓ* per price per unit time. The total limit order arrival rate is 2*νℓ*. Buy limit orders are uniformly distributed across the prices $P_{i}^{b}=\left\{a_{i}\left(t\right)-\nu ,a_{i}\left(t\right)-1\right\}$. Sell limit orders are uniformly distributed across the prices $P_{i}^{a}=\left\{b_{i}\left(t\right)+1,b_{i}\left(t\right)+\nu \right\}$. Market orders arrive at rate *m* per side per unit time making the total market order arrival rate 2*m*. Cancellations occur at a rate *c* per unit depth per unit time. The arrival rate of cancellations at price $\hat{p}$ is $c|X_{i}^{\hat{p}}\left(t\right)|$. The total arrival rate of cancellations at time *t* is $c(\sum_{p=1}^{n}|X_{i}^{p}\left(t\right)|)$.

### Multi-Market Model

The multi-market model is the process *Y*(*t*) defined in Eq (2), which models the simultaneous evolution of two limit order books. The index of price *p* in market 2 in the joint process is *p* + *n*. Events originate in each book according to the same rates as the single-market model, but events originating in one book may be routed to another based on the markets’ combined state. Market orders are routed with probability *α*. Limit orders are routed with probability *β*. Orders that are not routed execute or accrue in their home markets. The arrival rates for the multi-market model are summarized in Table 1.

$$Y\left(t\right)=\left(X_{1}\left(t\right)X_{2}\left(t\right)\right)=\left\{Y^{1}\left(t\right),\ldots ,Y^{2n}\left(t\right)\right\}\in Z^{2n}$$ (2)

**Table 1:** Aggregate arrival rates for the multi-market model.

Order	Side	Route	Rate
Limit	Bid	No	*ν*(1 − *β*)*ℓ*
Limit	Bid	Yes	*νβℓ*
Limit	Ask	No	*ν*(1 − *β*)*ℓ*
Limit	Ask	Yes	*νβℓ*
Market	Bid	No	(1 − *α*)*m*
Market	Bid	Yes	*αm*
Market	Ask	No	(1 − *α*)*m*
Market	Ask	Yes	*αm*
Cancelation	n/a	n/a	$c\sum_{i=1}^{2n}\left|Y^{i}\left(t\right)\right|$

The arrival rates for events in the multi-market model.

With arrival rates defined, we must connect order arrivals to updates of the state of the limit order book. Market orders decrease depth at the best bid and ask prices in their target markets. Limit orders increase depth in their target market at their specified price. Recall that bid depth is represented by a negative number, so a new limit order to buy at price $\hat{p}$ in market 2, which is not being routed, results in the state transition $y\to y-e^{\hat{p}+n}$, where *e*^*j*^ is the *j*th row of an identity matrix of size 2*n*.

The target markets are defined by the routing mechanisms which are as follows. Market orders are simply routed to the markets with the best opposite quote. If the opposite quotes are identical, the order remains in its originating market. The market order routing mechanism is described formally in Table 2. Limit order routing is more involved. Limit orders are classified into three groups: non-improving, improving and marketable. The routing of each type is treated differently. The classification and routing procedure for limit orders is provided in Table 3.

**Table 2:** Market order routing.

Side	Condition	Market
Bid	*a*_1_(*t*) < *a*_2_(*t*)	1
Bid	*a*_1_(*t*) > *a*_2_(*t*)	2
Bid	*a*_1_(*t*) = *a*_2_(*t*)	*h*
Ask	*b*_1_(*t*) > *b*_2_(*t*)	1
Ask	*b*_1_(*t*) < *b*_2_(*t*)	2
Askd	*b*_1_(*t*) = *b*_2_(*t*)	*h*

Market orders are simply routed to the market with the most favorable quote.

**Table 3:** Limit order classification.

Side	Price	Classification	Routing
Bid	$\hat{p}\leq \max\left\{b_{1}\left(t\right),b_{2}\left(t\right)\right\}$	Non-improving	Priority
Bid	$\max\left\{b_{1}\left(t\right),b_{2}\left(t\right)\right\}<\hat{p}<\min\left\{a_{1}\left(t\right),a_{2}\left(t\right)\right\}$	Improving	Ticks to opposite quote
Bid	$\min\left\{a_{1}\left(t\right),a_{2}\left(t\right)\right\}\leq \hat{p}$	Executable	Executing market
Ask	$\min\left\{a_{1}\left(t\right),a_{2}\left(t\right)\right\}\leq \hat{p}$	Non-improving	Priority
Ask	$\max\left\{b_{1}\left(t\right),b_{2}\left(t\right)\right\}<\hat{p}<\min\left\{a_{1}\left(t\right),a_{2}\left(t\right)\right\}$	Improving	Ticks to opposite quote
Ask	$\hat{p}\leq \max\left\{b_{1}\left(t\right),b_{2}\left(t\right)\right\}$	Executable	Executing market

Classification of limit orders for routing procedures.

**Fig 3:**
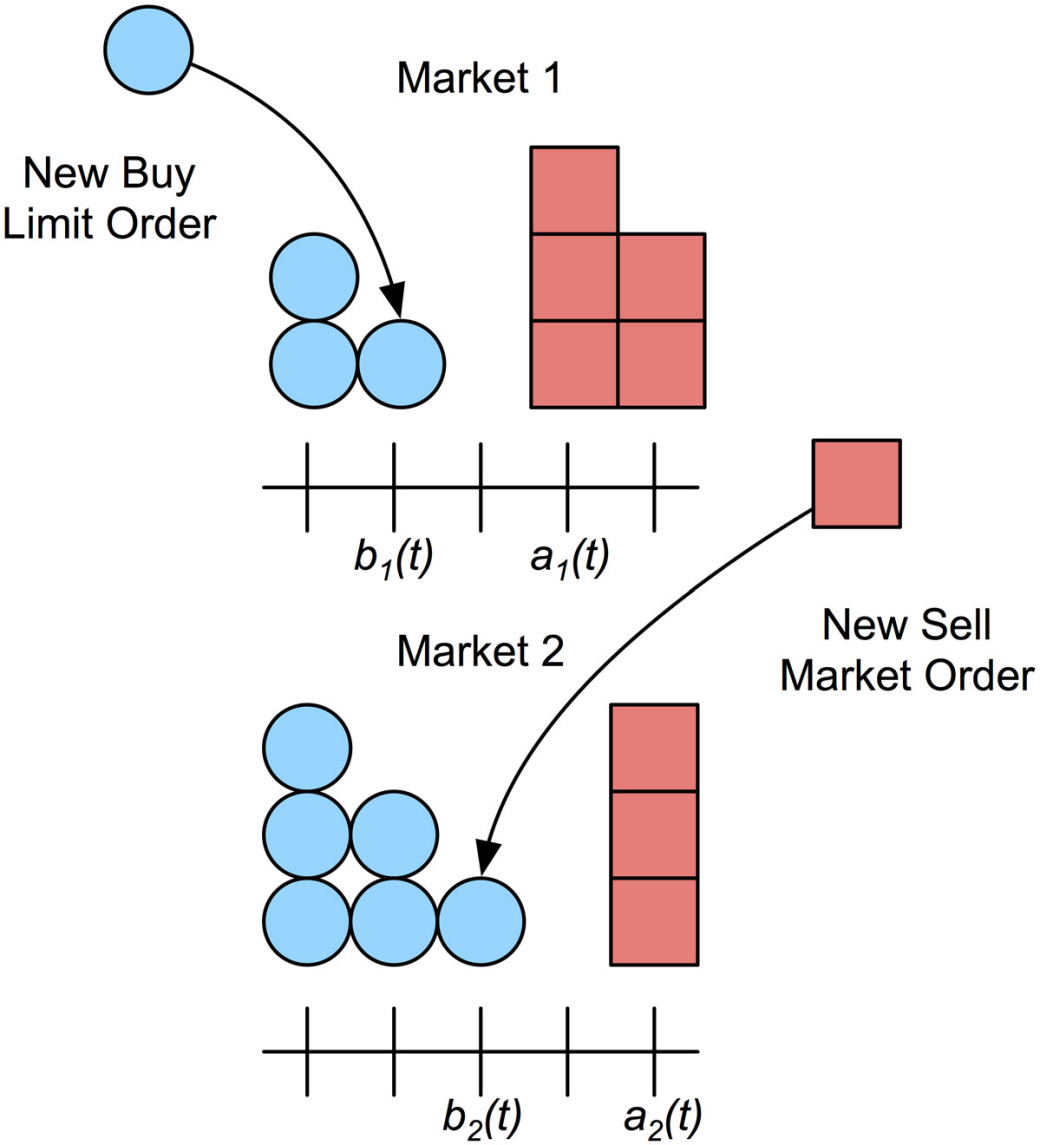
Order routing policy. A new buy limit order at price $\hat{p}=b_{2}\left(t\right)=b_{1}\left(t\right)+1$ arrives and is routed to market 1 since it will have better priority at that price, i.e. $d_{1}^{b}\left(\hat{p},t\right)<d_{2}^{b}\left(\hat{p},t\right)$. In market 1, a new limit order to buy at $\hat{p}$ is first in the queue with no depth ahead toward the best ask. In market 2, the same order is third in the queue. A new market order to sell is routed to market 2 since *b*_2_(*t*)>*b*_1_(*t*).

Non-improving limit orders are routed according to priority, which is defined as the depth between a new bid price $\hat{p}\in P_{i}^{b}\left(t\right)$ and the best ask in market *i* as defined by Eq (3). Analogously, the depth between a new ask price $\hat{p}\in P_{i}^{a}\left(t\right)$ and the best bid in market *i* is defined by Eq (4). A non-improving limit order at price $\hat{p}$ is routed to the market with the least depth between the new order and the opposite quote. The routing for non-improving limit orders is given in Table 4. The routing of a non-improving limit order is illustrated in Fig 3.

$$d_{i}^{b}\left(\hat{p},t\right)=\sum_{p=\hat{p}}^{a_{i}\left(t\right)}\left|X_{i}^{p}\left(t\right)\right|$$ (3)

$$d_{i}^{a}\left(\hat{p},t\right)=\sum_{p=b_{i}\left(t\right)}^{\hat{p}}\left|X_{i}^{p}\left(t\right)\right|$$ (4)

**Table 4:** Non-improving priority routing mechanism.

Side	Condition	Destination
Bid	$d_{1}^{b}\left(\hat{p},t\right)<d_{2}^{b}\left(\hat{p},t\right)$	1
Bid	$d_{1}^{b}\left(\hat{p},t\right)>d_{2}^{b}\left(\hat{p},t\right)$	2
Bid	$d_{1}^{b}\left(\hat{p},t\right)=d_{2}^{b}\left(\hat{p},t\right)$	*h*
Ask	$d_{1}^{a}\left(\hat{p},t\right)<d_{2}^{a}\left(\hat{p},t\right)$	1
Ask	$d_{1}^{a}\left(\hat{p},t\right)>d_{2}^{a}\left(\hat{p},t\right)$	2
Ask	$d_{1}^{a}\left(\hat{p},t\right)=d_{2}^{a}\left(\hat{p},t\right)$	*h*

Routing procedures for non-improving limit orders. Limit orders originating with price $\hat{p}$ are routed according to the conditions in the table where *h* indicates the originating market.

Improving limit orders are routed according to the distance in ticks to the opposite quote. The routing of improving limit orders is provided in Table 5. If the distance to the opposite quote is 0 in one of the two markets, then the order is marketable and will execute in that market.

**Table 5:** Limit order routing for improving orders.

Side	Condition	Destination
Bid	$a_{1}\left(t\right)-\hat{p}<a_{2}\left(t\right)-\hat{p}$	1
Bid	$a_{1}\left(t\right)-\hat{p}>a_{2}\left(t\right)-\hat{p}$	2
Bid	$a_{1}\left(t\right)-\hat{p}=a_{2}\left(t\right)-\hat{p}$	*h*
Ask	$\hat{p}-b_{1}\left(t\right)<\hat{p}-b_{1}\left(t\right)$	1
Ask	$\hat{p}-b_{1}\left(t\right)>\hat{p}-b_{1}\left(t\right)$	2
Ask	$\hat{p}-b_{1}\left(t\right)=\hat{p}-b_{1}\left(t\right)$	*h*

Classification of limit orders for routing procedures.

### Simulation Procedure

The procedure for simulating the model is based on the standard stochastic simulation algorithm, also known as the Gillespie algorithm [25]. An outline of the algorithm is as follows.

1. Initialize the state *Y*(*t*) at *t* = 0 and select a duration *T* for the simulation to run.
2. Denote the arrival rates *λ*_*i*_(*t*) for all possible events *i* = 1…*m*.
3. Calculate the sum of the transition rates $\lambda \left(t\right)=\sum_{i}^{m}\lambda_{i}\left(t\right)$.
4. Simulate the time, *τ*, until the next transition by drawing from an exponential distribution with mean 1/λ(t).
5. Choose the transition type by drawing from a discrete distribution where the probability of event *i* is $\lambda_{i}\left(t\right)/\lambda \left(t\right)$.
6. Update the process according to the interarrival time and transition type.
7. Repeat steps 2–6 until *t* > *T*.

**Fig 4:**
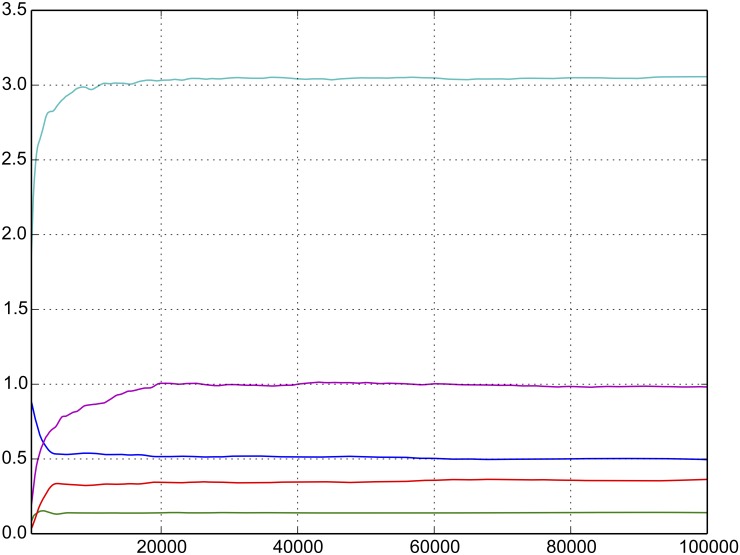
Welch’s graphical method for identifying the warm-up period for the simulation.

The model is initialized with a depth of 5 lots at the best *ν* prices on each side of the market. The best bid and best ask in each market is set to ⌊n / 2⌋ and ⌊n / 2⌋ + 1, respectively. For all replications, the model is warmed up to avoid initialization bias. We use Welch’s graphical method to determine the warm-up period [26]. In brief, the method consists of averaging over replications and then examining a moving average of the various output quantities. Fig 4 displays the moving average of several output quantities for *α* = 0.05 and *β* = 0.05. The output is based on 10 replications of approximately 100,000 epochs. The values are averaged at each epoch and the moving average is calculated with respect to the last 1000 observations. Using this approach, we end the initial warm up period at *t*_*s*_ = 650, which is equivalent to approximately 100,000 epochs.

The embedded Markov chain associated with *Y*(*t*) is simulated by normalizing the arrival rates of individual events by *λ*(*t*) given in equation Eq (5), which is the sum of the rates given in Table 1. At each discrete epoch, the clock is advanced according by *τ*, which is drawn from an exponential distribution with mean 1 / *λ*(*t*), and the state is updated according to the model. The model terminates when *t* > *T*. Statistics are calculated for *t*_*s*_ < *t* < *T* where *T* = 1650.

$$\lambda \left(t\right)=4v\ell +4m+c\sum_{p=1}^{2n}\left|Y^{p}\left(t\right)\right|$$ (5)

## Results and Discussion

The main results are summarized by response surfaces for crossed and locked states as *α* and *β* are varied between 0 and 1. We also report on mean spread and volatility of the market as defined by the consolidated books. The results are based on 30 replications of length *T* − *t*_*s*_ = 1000 for each parameterization of *α* and *β*. We also report on the sensitivity of the main results to 10% changes in the arrival of market and limit order parameters *ℓ* and *m*. We expect some sensitivity as these two parameters drive the spread and volatility of the single market model.

Gatheral and Oomen (2010) use the single market model to evaluate alternative procedures for computing realized variance. They use parameters *ℓ* = 1, *m* = 5, *c* = 0.2, *ν* = 10, which they find results in a microstructure noise ratio similar to that of stocks in the DJ30. Our main results are estimated with parameters *ℓ* = 1, *m* = 5, *c* = 0.2, *ν* = 20, which results in an average depth in each market of 150 lots. Increasing the band *v* from 10 to 20 results in a higher mean depth across the markets, which also results in additional stability. With *ν* = 20, neither side of the book emptied out across all of our replications. Earlier simulations with *ν* = 10 were not stable for some values of *α* and *β*, which resulted in extreme price moves when the book emptied out on either the buy or sell side. The model, in general, is stable over a wide range of the parameters. Our goal is not to replicate the stylized facts of any particular market, but to study the effects of order routing under reasonable assumptions of order flow, spread and volatility.

The size of the price grid is *n* = 10,000, which is large enough that results are not affected by the bounds of the grid. We present the main results as response surfaces and then report on a sensitivity analysis of order flow parameters. Specific parameters, replications and other simulation details are given alongside the results.

### Order Routing Results

**Fig 5:**
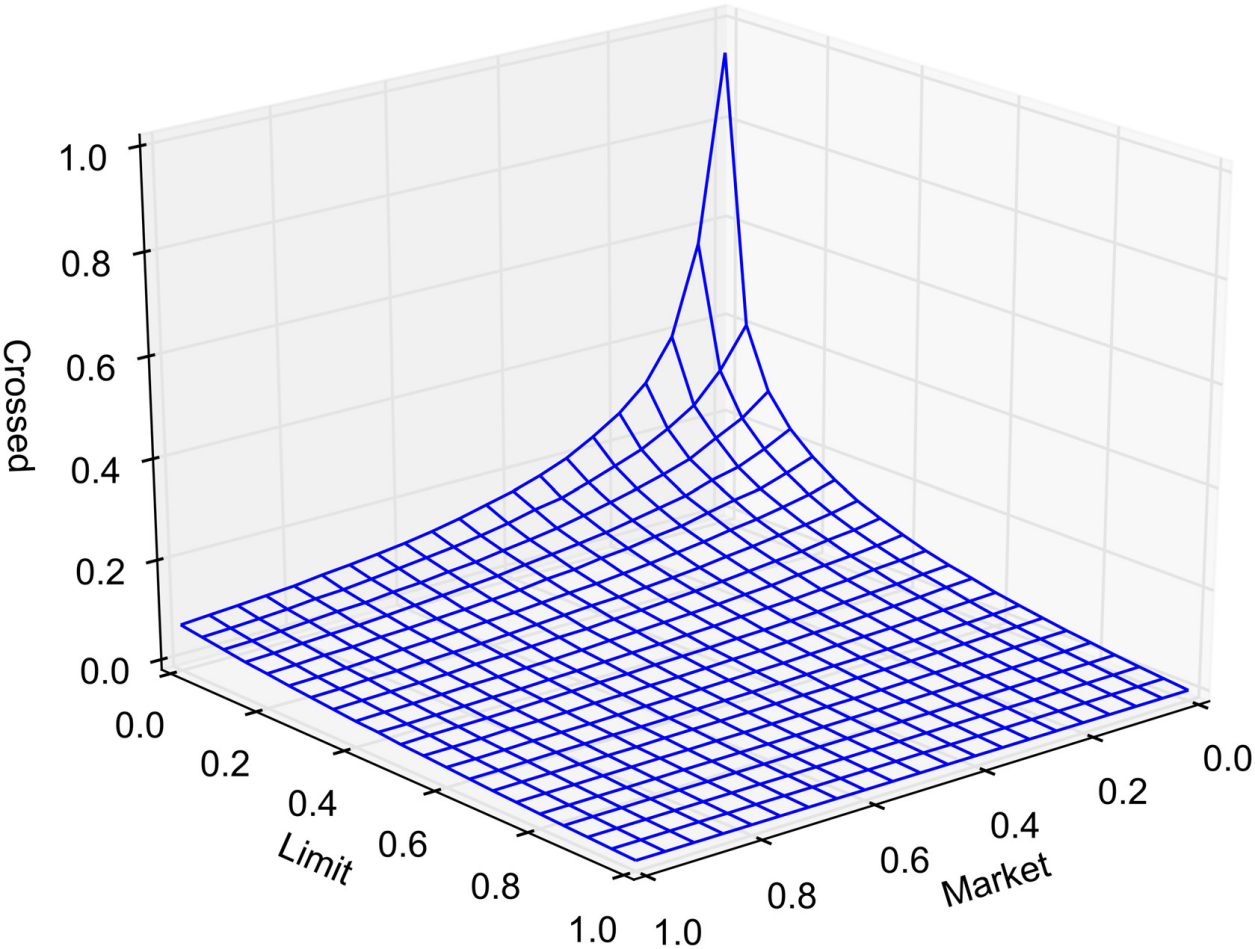
Percentage of time markets are crossed.

**Fig 6:**
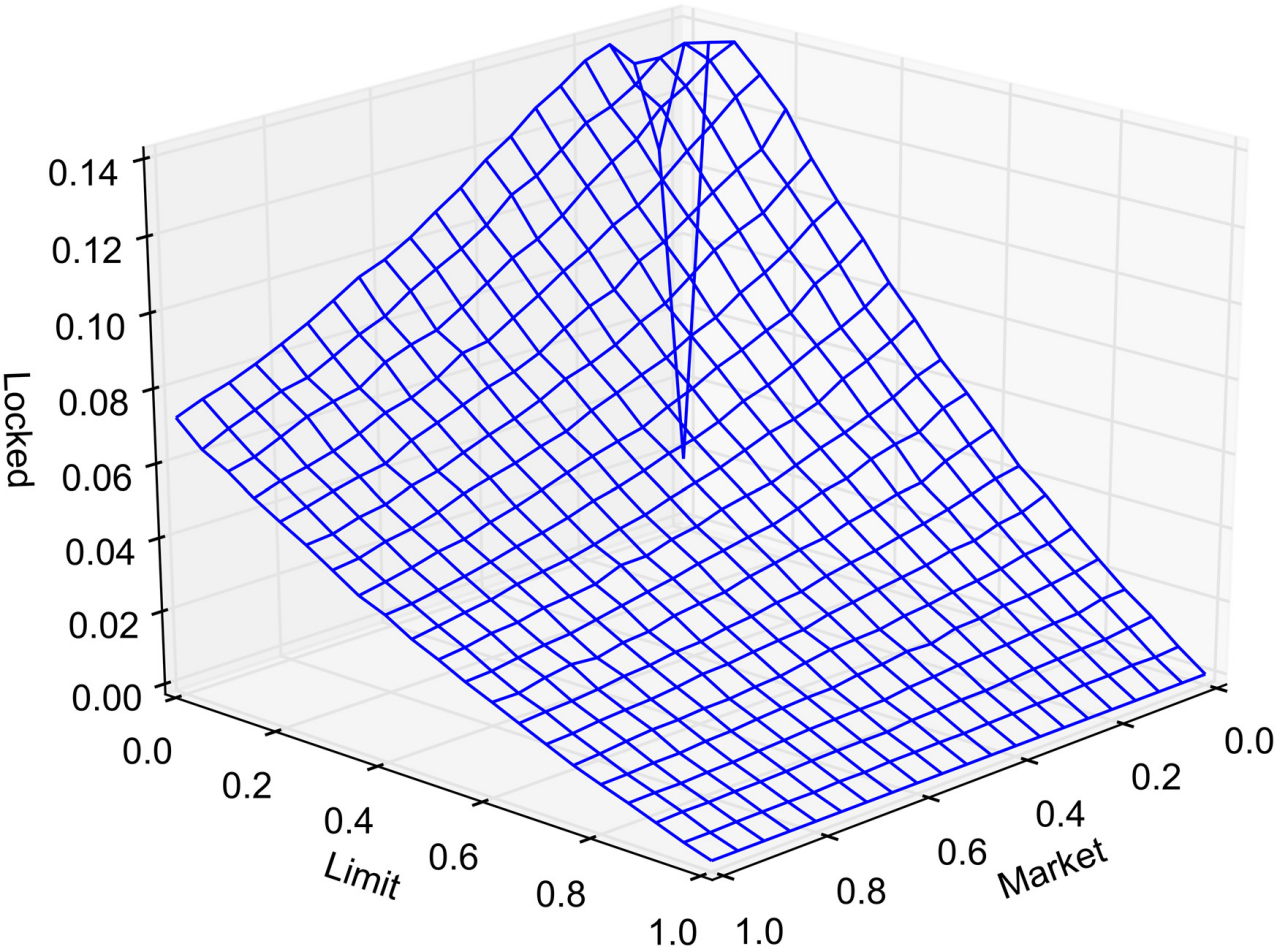
Percentage of time markets are locked.

**Fig 7:**
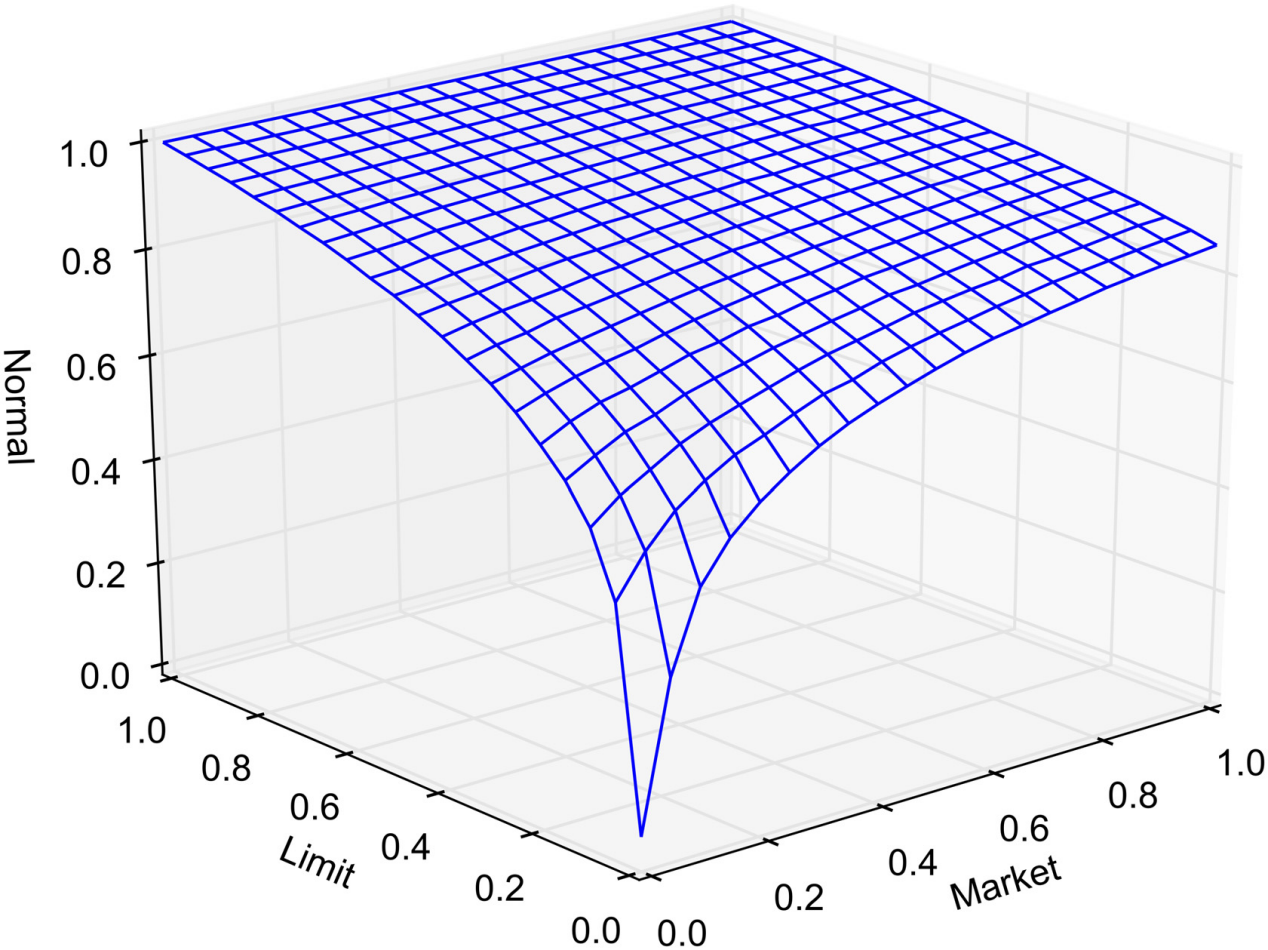
Percentage of time markets are normal (neither crossed nor locked).

The probability of market routing and limit routing, as defined earlier, is varied between 0 and 1 by increments of 0.05. At any given time, the state of the market is classified as either crossed, locked or normal. The average portion of time that the market spends in a crossed, locked or normal state, as a function of market and limit order routing, is reported in Figs 5, 6 and 7, respectively. Each point in the lattice of the wireframe represents the mean of the outputs across 30 replications. Crossed and locked market durations fall rapidly as routing is increased. Markets are locked 7.2% of the time and crossed 7.1% of the time when *α* = 1 and *β* = 0. When *β* = 1, all limit orders are routed eliminating the possibility of crossed or locked markets.

**Fig 8:**
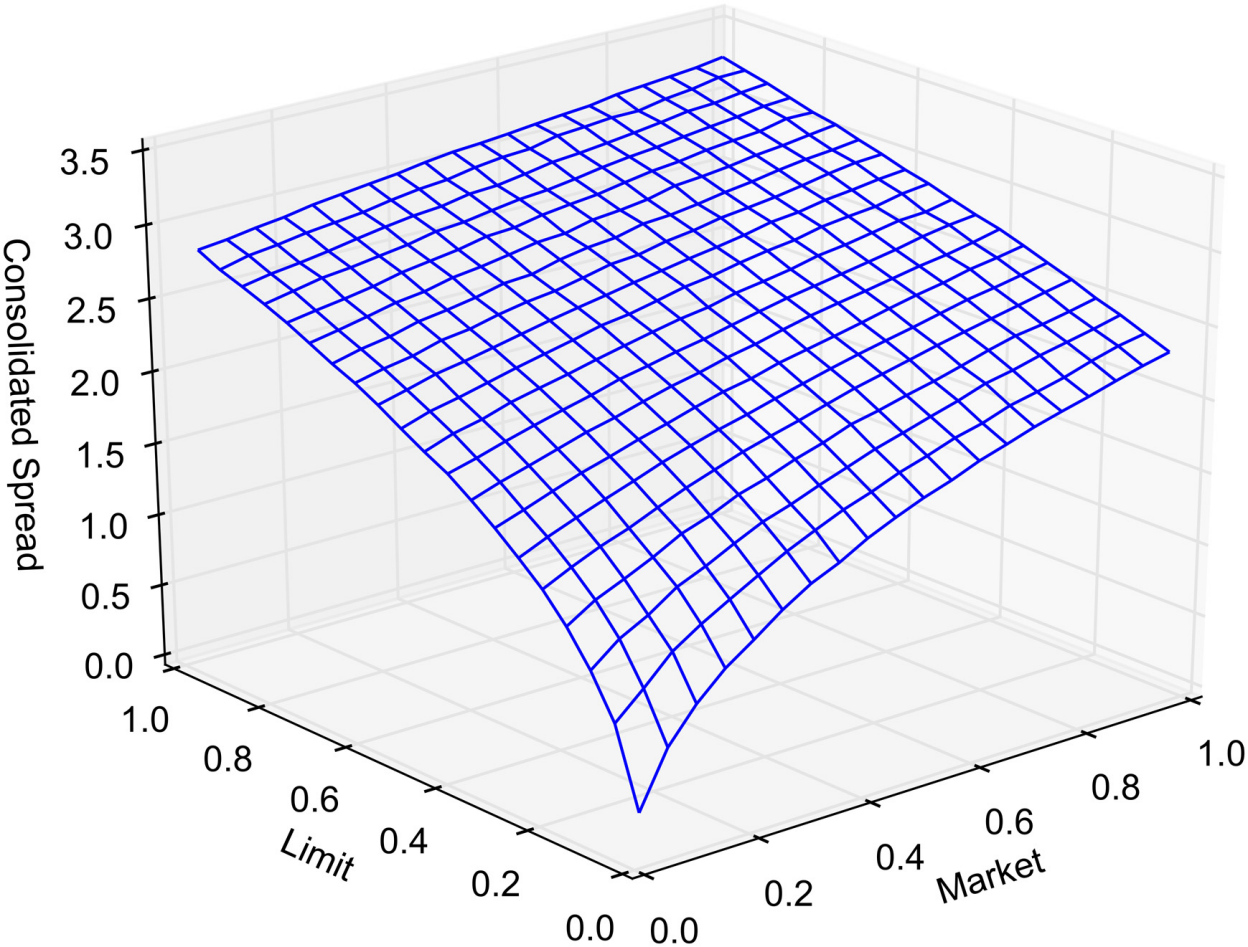
Mean spread in the consolidated book.

Fig 8 reports the mean spread of the consolidated order book, i.e. min{*a*_1_(*t*), *a*_2_(*t*)} − max{*b*_1_(*t*), *b*_2_(*t*)}. Note that the spread in the consolidated book can be negative when *β* ≠ 1. Order routing increases the mean consolidated spread by eliminating episodes where it is non-positive. Consider, however, the case when *β* = 1, where the consolidated spread is strictly positive. The spread is increasing with *α* since routed market orders are more likely to affect the best quotes.

**Fig 9:**
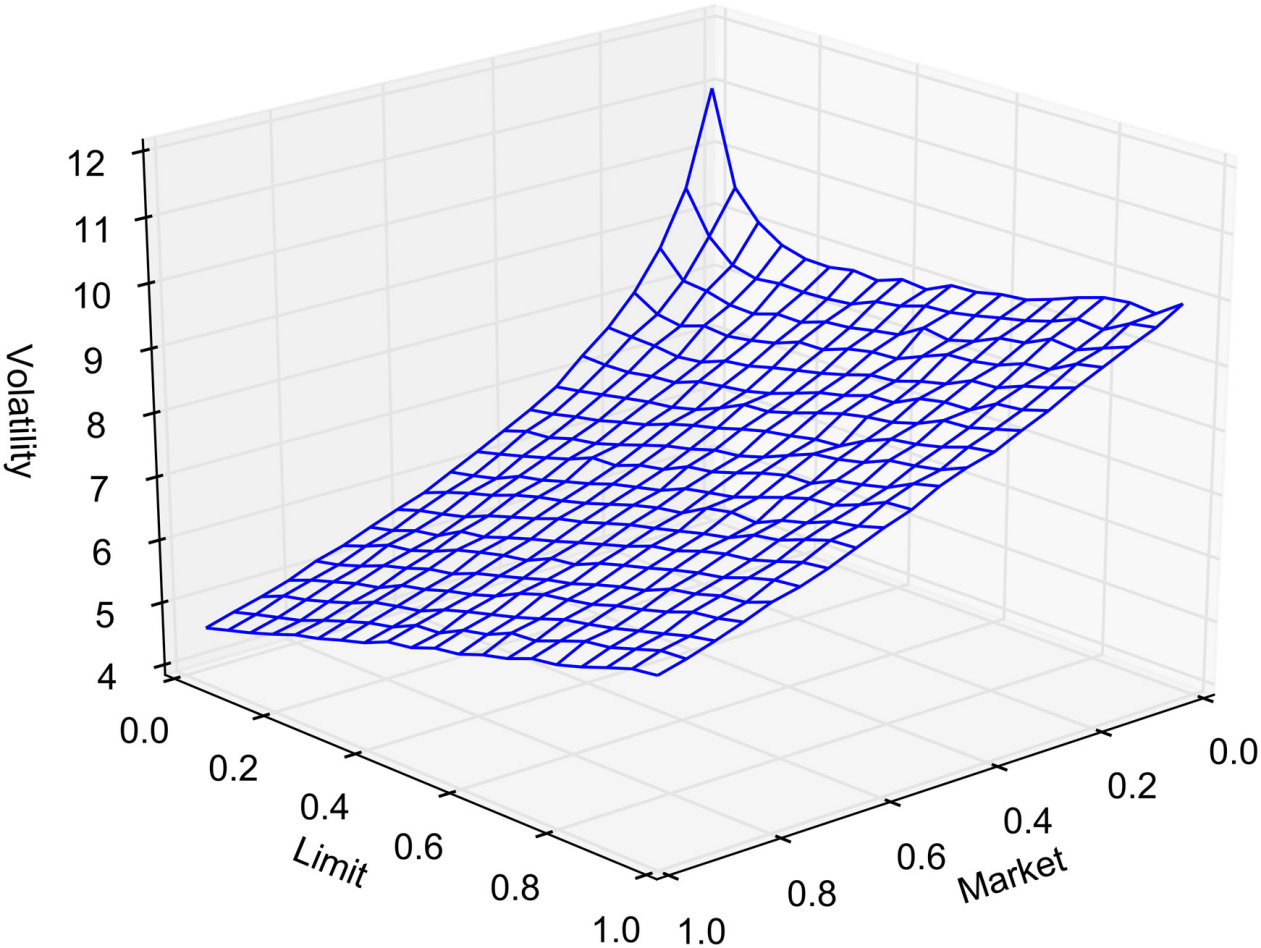
Volatility as measured by the variance of the difference in transaction prices.

Volatility, like the other statistics we report, is calculated online. We define volatility to be the variance of the differences in transaction prices. The variance is computed using the well-known recursion given in Eqs 6 and 7 where *μ*_1_ = *x*_1_ and *ϕ* = 0 [27]. The *k*th transaction price is denoted *x*_*k*_. When the simulation terminates the variance is given as *ϕ* / (K − 1) where *K* is the total number of transactions. Market order routing decreases volatility as expected. Routed market orders execute at the best bid and ask in the consolidated book, which is by definition a smaller price range. When all market orders are routed to the best quotes (*α* = 1), there appear to be two competing effects when limit order routing is increased. With relatively few routed orders, an increase draws the market nearer, whereas later, more routing increases the spread, resulting in higher price volatility. Fig 9 reports volatility as a function of *α* and *β*. Spread and volatility results for several discrete cases are reported in Table 6.

$$\mu_{k}=\mu_{k-1}+\left(x_{k}-\mu_{k-1}\right)/k$$ (6)

$$\phi_{k}=\phi_{k-1}+\left(x_{k}-\mu_{k-1}\right)\left(x_{k}-\mu_{k}\right)$$ (7)

**Table 6:** Consolidated spread and volatility results.

Routing		Spread		Volatility	
*α*	*β*	Mean	Std. Dev.	Mean	Std. Dev.
1	1	3.25	0.04	6.8	0.20
0	1	2.76	0.03	10.3	0.23
1	0	2.12	0.02	4.6	0.13
0.5	0.5	2.46	0.03	7.3	0.16

Replication results based on 30 replications with *ℓ* = 1, *m* = 5, *c* = 0.2, *v* = 20, *n* = 10,000. The spread is reported for the consolidated order book.

### Sensitivity Analysis

**Fig 10:**
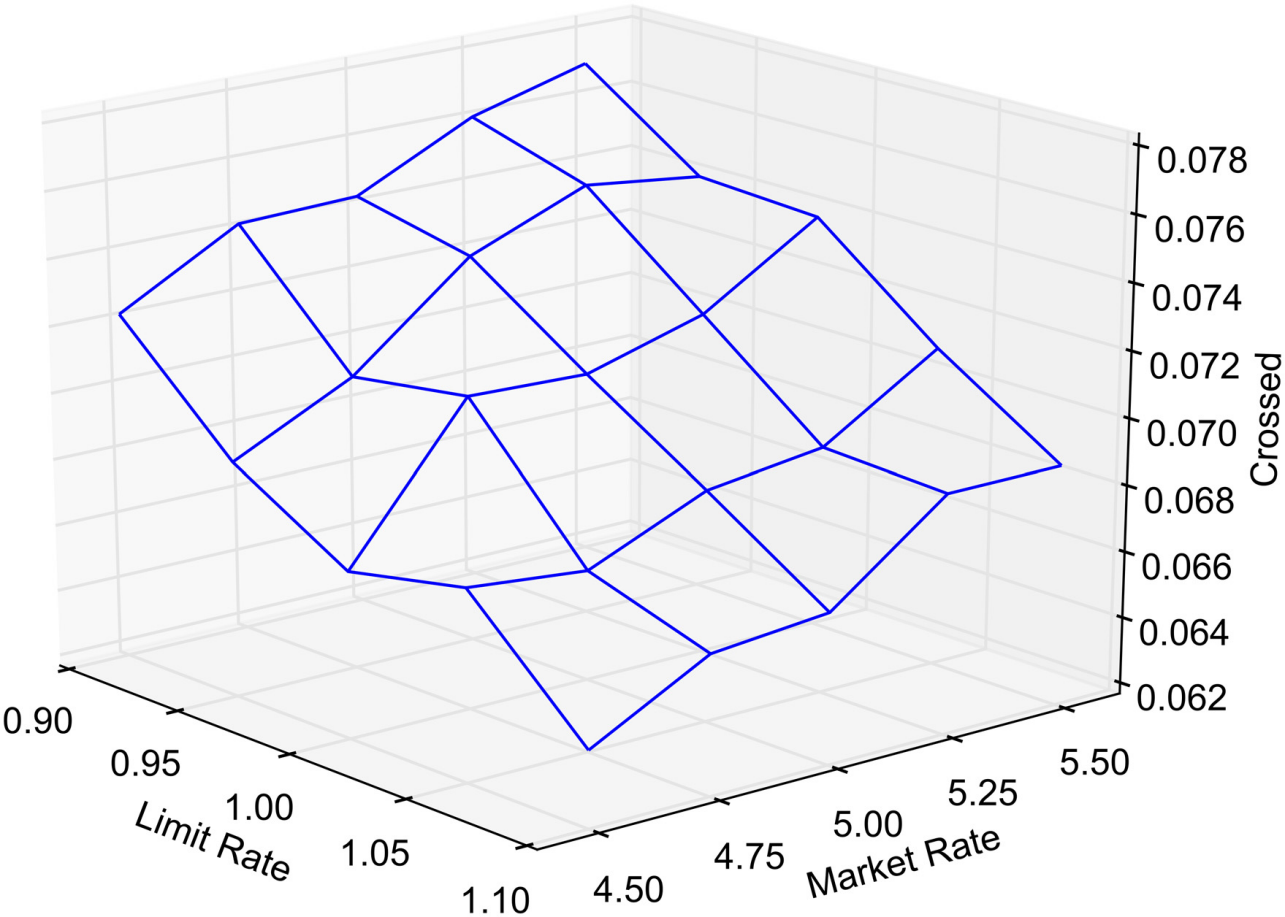
Sensitivity of crossed state duration. Proportion of time market is in crossed state as a function of limit and market order arrival rates for *α* = 1 and *β* = 0.

**Fig 11:**
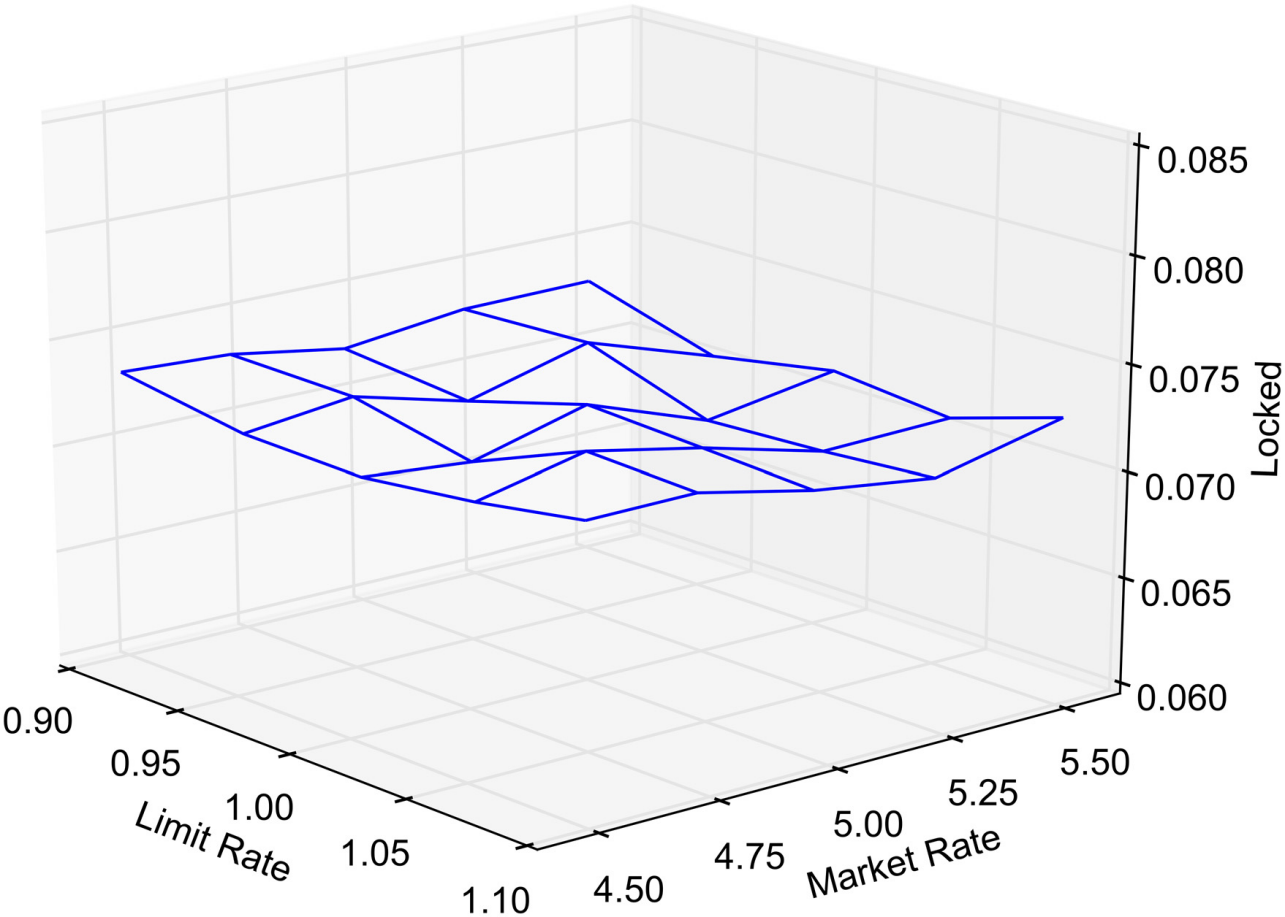
Sensitivity of locked state duration. Proportion of time market is in locked state as a function limit and market order arrival rates for *α* = 1 and *β* = 0.

**Fig 12:**
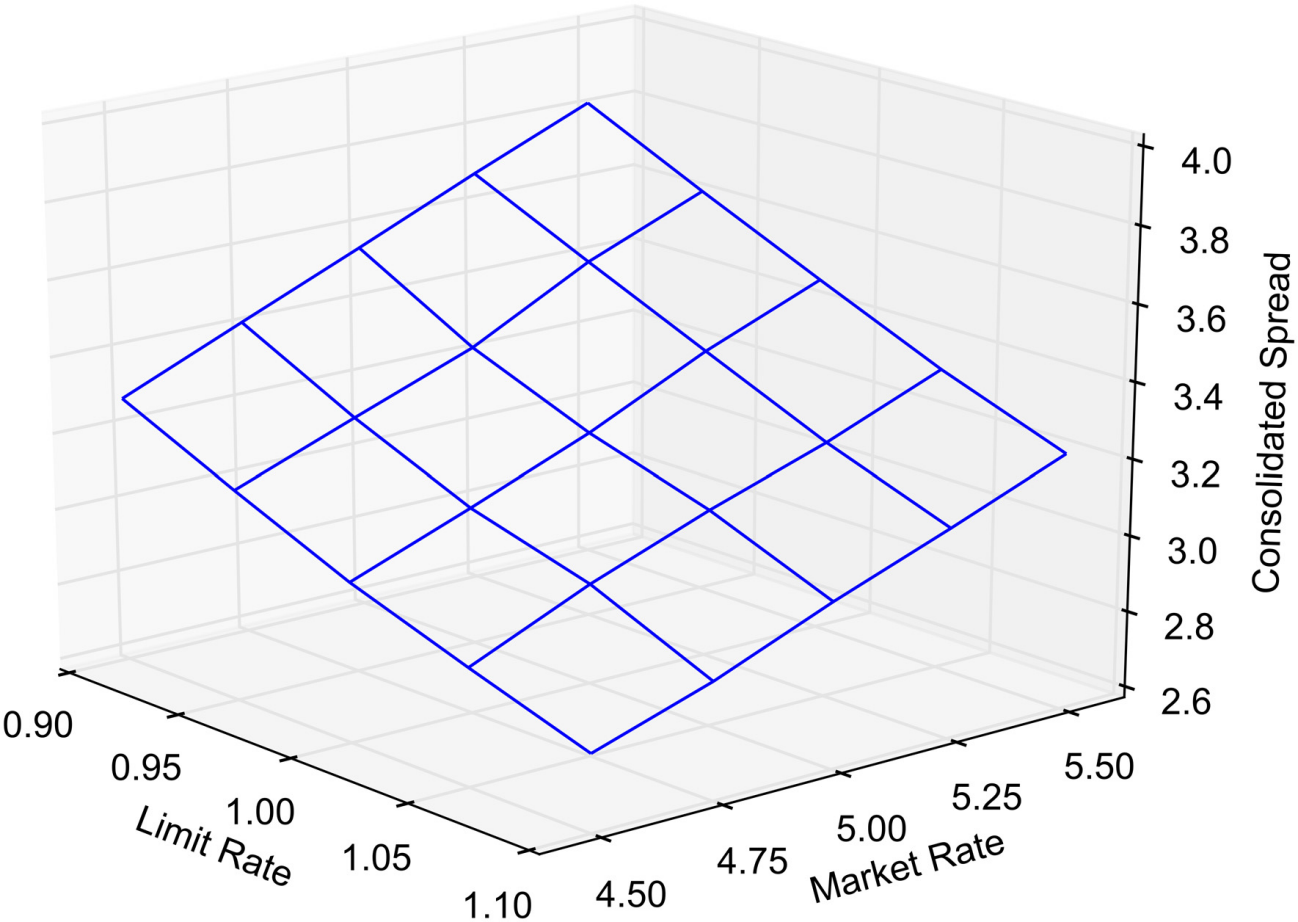
Sensitivity analysis of the consolidated spread. Mean consolidated spread as market and limit order arrival rates are varied for *α* = 1 and *β* = 1.

**Fig 13:**
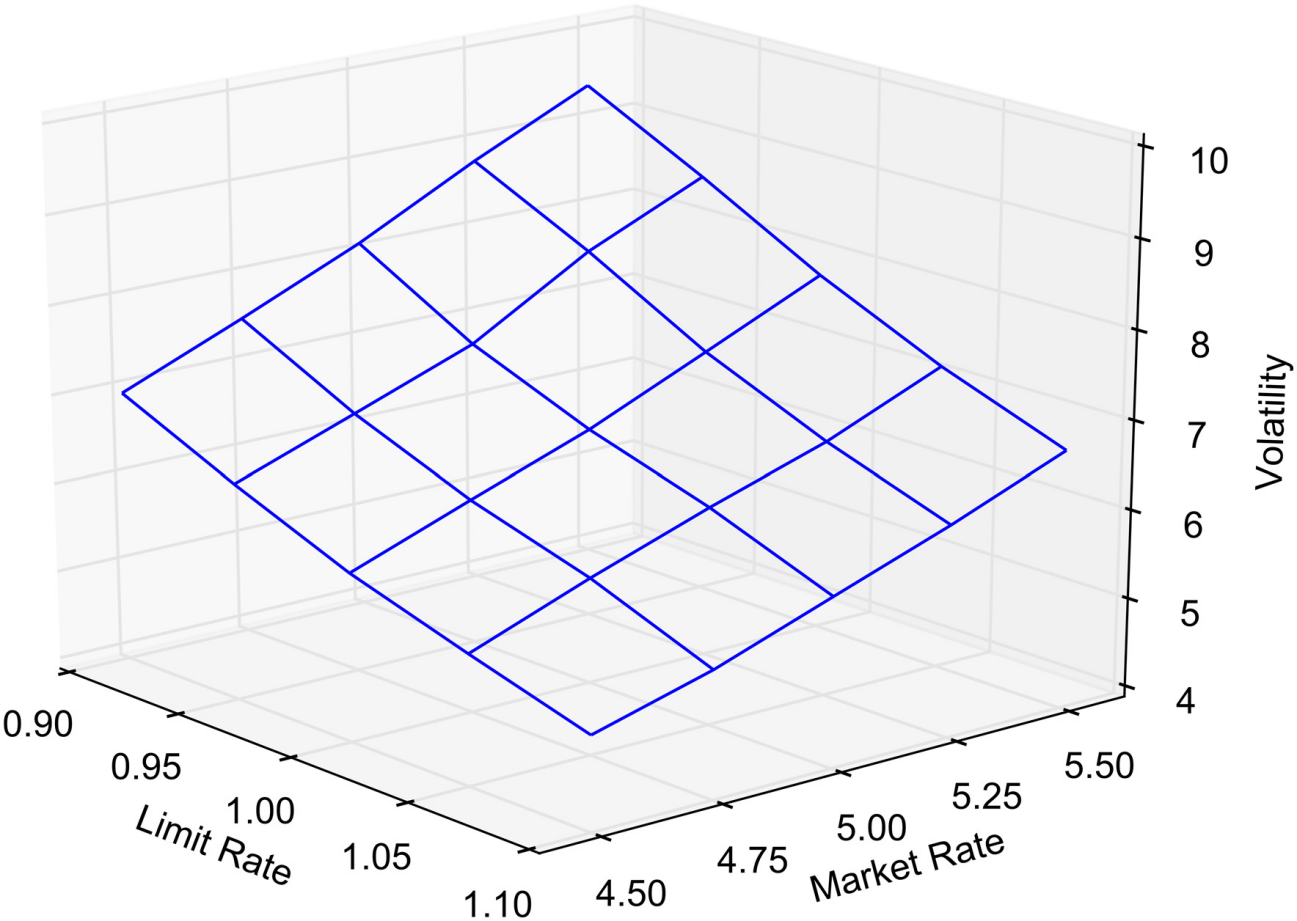
Sensitivity analysis of volatility. Volatility of transaction prices as a function limit and market order arrival rates for *α* = 1 and *β* = 1.

The crossed and locked state durations, as well as spread and volatility, show some sensitivity to the main parameters of the market. This is to be expected as limit and market order arrival rates drive the characteristics of the single market model. For each of the model outputs reported in the main results, we provide a response surface as the market and limit arrival rates are varied by ±10%. Figs 10 and 11 report on crossed and locked state durations for *α* = 1 and *β* = 0. Figs 12 and 13 report the sensitivity of spread and volatility to changes in market and order arrival rates for *α* = 1 and *β* = 1.

## Conclusions

The zero-intelligence approach is a powerful modeling framework for examination of fragmented markets. By modeling order routing as part of the market mechanism, we maintain a parsimonious model of order-driven markets that has the potential to explain stylized facts beyond crossed and locked markets. The model helps develop intuition for the effects of simple order routing procedures on the statistical properties of a multi-market system. The results also provide a benchmark against which more complex agent-based models may be compared.

Empirical validation is also a possibility since all model parameters, including *α* and *β*, could potentially be estimated from data. The model has an interpretation in terms of market demographics as well. For example, parameterizations of our model can be interpreted as a markets consisting of a certain percentage of participants with superior market access. We believe models of this type are uniquely capable of examining complex market structures while simultaneously accounting for market demographics. The model has the flexibility to be extended in a number of directions, including examination of markets with asymmetric trading volume and more complex market structures.

## Supporting Information

S1 Dataset Main results. Simulation output and summary files for main results.
(ZIP)

S2 Dataset Sensitivity analysis. Simulation output and summary files for the sensitivity analysis.
(ZIP)
